# Targeted Strategies for Treatment of Colorectal Cancer: Exploring the Use of Aptamers and Gold Nanoparticles

**DOI:** 10.3390/molecules31183150

**Published:** 2026-09-08

**Authors:** Bolu Bimbola Oladunjoye, Nicole Remaliah Samantha Sibuyi, Miché Desline Meyer, Mervin Meyer, Abram Madimabe Madiehe

**Affiliations:** 1 Nanobiotechnology Research Group, Department of Biotechnology, University of the Western Cape, Private Bag X17, Bellville 7535, South Africa; 3919192@myuwc.ac.za; 2 Department of Science, Technology and Innovation (DSTI)/Technology Innovation Agency Nanotechnology Platform, Department of Biotechnology, University of the Western Cape, Private Bag X17, Bellville 7535, South Africa; 3135272@myuwc.ac.za (M.D.M.); memeyer@uwc.ac.za (M.M.); 3 DSTI/National Research Foundation SARChI Chair: Sustainable Nanotechnology, Department of Biotechnology, University of the Western Cape, Private Bag X17, Bellville 7535, South Africa; 4 South African Medical Research Council (SAMRC)/UNIVEN/Mintek Digital Health and AI for Occupational Health-Mining Sector Research Unit, Advanced Materials Division, Mintek, Private Bag X3015, Randburg 2125, South Africa

**Keywords:** aptamers, colorectal cancer, drug delivery, gold nanoparticles, targeted therapy

## Abstract

Colorectal cancer (CRC) is the second leading cause of cancer-associated mortality and accounts for ~10% of all cancer cases. Global CRC cases were estimated at 1.9 million and 930,000 deaths in 2022. Despite improved access to CRC screening and treatment, death rates are expected to drastically increase in low-resource areas, highlighting an urgent need for innovative CRC treatment interventions. Current chemotherapy presents challenges, such as poor bioavailability, multidrug resistance, and adverse bystander effects. Therefore, it is imperative to develop new and CRC-specific therapeutic strategies. Targeted therapy has significantly improved drug delivery and efficacy in the treatment of various diseases, including cancer. Although several preclinical studies are currently ongoing in cancer therapy, there is a paucity of information about targeted CRC treatment. Hence, this review highlights the promising role of aptamer- and gold nanoparticles (AuNPs)-based systems for targeted CRC treatment. This strategy leverages the unique properties of aptamers and AuNPs as drug carriers, targeting and therapeutic agents. Overall, aptamers in combination with AuNPs are projected to improve the delivery of chemotherapeutics to CRC cells. However, further studies are warranted to validate the biodistribution and safety profile of aptamer- and AuNPs-based systems as potential tools for CRC treatment.

## 1. Introduction

Cancer is a debilitating disease that has taken its toll on human health and the economy. It remains a public health threat that continues to plague individuals, families and health systems worldwide. Colorectal cancer (CRC) is among the leading cancer types with high mortality. On the global scale, there were 1.9 million new cases and about 930,000 deaths reported for CRC in 2022 [1]. This cancer type is more prevalent in individuals aged 50 years and older; however, in recent years, the disease is increasingly being diagnosed in young adults and adolescents [1]. Major risk factors for CRC include age, gender, lifestyle and hereditary factors [2]. Conventional treatment strategies include chemotherapy, surgery, radiotherapy, and immunotherapy [3]. Among these, chemotherapy is the first line of intervention and the primary treatment in advanced CRC. Chemotherapeutics used in the clinical management of CRC include 5-fluorouracil (5-FU), doxorubicin (dox), irinotecan, capecitabine, oxaliplatin, leucovorin, levamisole and methotrexate [4]. However, conventional treatment strategies are associated with several limitations, including high treatment costs, limited drug penetration into the tumor microenvironment, as well as inadequate selectivity between cancerous and non-cancerous cells. These limitations can result in significant systemic toxicity and adverse effects, including nausea, vomiting, alopecia, myelosuppression, immunosuppression, and oedema [5].

Advances in targeted therapy have improved target specificity, enhanced drug delivery and efficacy. In this instance, antibodies directed against specific targets are used to either deliver chemotherapy or recruit immune cells to kill diseased cells. Registered antibody-based therapies used in CRC include panitumumab (Vectibix^®^), cetuximab (Erbitux^®^), which target the epidermal growth factor (EGF) receptor, and bevacizumab (Avastin^®^), which targets vascular endothelial growth factor (VEGF) [6]. However, antibodies can interact with the immune system, resulting in immunotoxicity. In recent years, aptamers are being explored as alternatives to antibodies, as they have similar biological properties and are stable at high temperatures [7]. This review explores aptamer- and AuNPs-based therapeutic strategies for CRC, highlighting their potential to enhance the targeted delivery of chemotherapeutic agents, reduce off-target toxicity and improve therapeutic outcomes. 

Nanotechnology-based interventions have been exploited in targeted drug delivery to ensure site-specific delivery, adequate toxicity to cancer cells, and biocompatibility. Nanotechnology involves the use of nanomaterials, which are classified as organic and inorganic NPs. The organic NPs consist of liposomes, micelles, and polymers, while inorganic NPs are mostly metallic NPs (MNPs) include gold, silver, iron, zinc, and copper NPs [8]. Nanomaterials have unique physical and chemical properties, such as small size, surface composition, and shape, which enhance their activities [9]. Their small size assists in their cellular uptake, eliciting their effect through either passive or active targeting. In passive targeting, NPs evade the reticuloendothelial system (RES) and accumulate in diseased cells, while active targeting involves the use of targeting moieties (peptides, aptamers and antibodies) to facilitate the uptake of NPs by diseased cells [10].

AuNPs have shown promise in disease therapy owing to their small size, ease of synthesis and surface functionalization, surface plasmon resonance (SPR), and low cytotoxicity. Hence, AuNPs have been explored in the treatment of diseases such as cancer [11], microbial infections [12], and diabetic nephropathy, among others [13]. AuNP-based treatments in clinical trials include Aurolase used for the treatment of prostate cancer and other solid tumors, naNocovid and naNodengue for the prevention of COVID-19 and dengue infections, respectively [14]. In cancer therapy, AuNPs serve as drug delivery systems, drug sensitizers and photothermal agents [15,16]. AuNPs were used as delivery vehicles for anticancer drugs such as cisplatin [17], dox [18], paclitaxel [19] and methotrexate [20]. As drug carriers, AuNPs improved cellular uptake in cancer cells and exerted antitumor effects in mice xenografts [18,21]. Additionally, the photothermal properties of AuNPs were exploited in the treatment of colorectal [22], breast [11], prostate [23], and hepatic [24] cancers. When used in combination therapy, AuNPs enhanced drug efficacy in CRC cells at non-lethal concentrations and improved biocompatibility [25]. However, AuNPs facilitate cellular uptake through passive targeting and localize into non-target cells, leading to systemic side effects such as neurotoxicity, cardiotoxicity and mucositis [26]. To forestall this challenge, aptamers can be conjugated to AuNPs as targeting molecules to ensure the efficient delivery of therapeutics to cancer sites [21,27].

## 2. Cancer Burden and Epidemiology

Cancer poses a significant health threat worldwide. In 2022, the disease accounted for about 20 million new cases and 9.7 million deaths. These cases were mainly attributed to inadequate access to early diagnosis and treatment [22]. Globally, cancer is the second most common cause of death following ischemic heart disease. The disease is a leading cause of deaths in individuals between the ages of 45 and 64 years and is very costly to manage [28]. In the next two decades, new cases are estimated to increase to 24 million, while 40% of individuals may be diagnosed with cancer during their lifetime [29].

Cancer is a multifactorial disease whose cause has been attributed to genetic mutations that occur during cell division, prolonged exposure to environmental contaminants and inherited genetic factors [30]. Other risk factors that predispose humans to cancer include hormones, immune disorders, obesity, sedentary lifestyle, unhealthy diet, alcohol, tobacco [31,32], and exposure to carcinogens such as aflatoxins, drugs, benzene, asbestos, organophosphates, carbon tetrachloride, polychlorinated biphenyls, and arsenic. Infectious diseases also increase the risk of cancer; for example, hepatitis B virus contributes to liver cancer, human papillomavirus to cervical cancer, *Helicobacter pylori* to gastric cancer, while human immunodeficiency virus, Epstein–Barr virus and hepatitis C are also risk factors for other types of cancer [32,33].

The development of cancer is characterized by uncontrollable cell proliferation, the evasion of apoptosis, increased angiogenesis, malignancy, tissue invasion and metastasis. These hallmarks represent properties that a normal cell acquires to become tumorigenic and malignant [34,35]. Normal cells can control the production and release of growth-promoting signals, while cancer cells proliferate nonstop. Normal cells undergo programmed cell death when there is an irreparable error during DNA replication. In contrast, cancer cells are insensitive to apoptotic signals, unresponsive to growth suppressor genes, and evade apoptosis [35]. As such, cancer cells develop mechanisms that inactivate or suppress growth signals that regulate their proliferation, limiting senescence and apoptosis [36]. They express factors that extend neovasculature, ensuring an adequate supply of nutrients and oxygen. Under normal physiological conditions, cells undergo senescence and die after a certain number of divisions, whereas cancer cells become immortal and replicate continuously without senescence [36]. They eventually spread or metastasize to other sites where space, oxygen, and nutrients are available [34]. As a result, there are over 100 cancer types, which are all named based by the organ or tissue where they are localized. CRC is among the top three types of cancer reported to have high prevalence and mortality rates, and it is the focus of this review. The death rates of CRC are projected to increase by 93.5% in 2050 [37], which raises a serious concern as CRC is challenging to treat when diagnosed at advanced stages (Stages III and IV). Therefore, this warrants further investigations into effective, targeted and sustainable therapeutic strategies for CRC management.

### CRC and Risk Factors

CRC is the third most prevalent cancer and second leading cause of cancer-related deaths worldwide. The disease remains a global challenge with approximately 1.9 million new cases and 930,000 deaths that were estimated in 2022 [22]. This disease accounts for nearly 10% of all cancer cases and occurs mainly in individuals aged 50 years and above, although it has also been reported in younger individuals. By 2040, the global burden of CRC is expected to increase to 3.2 million cases (63% increase) and 1.6 million fatalities (73% increase) per annum [1]. Although the incidence rate and deaths have reduced in high-income countries due to adequate screening tests and easier access to treatments, the overall 5-year survival rate is still less than 60%, and the prognosis of the disease is dependent on early diagnosis [38]. The early diagnosis of CRC is associated with higher survival rates, while advanced CRC has lower survival rates [39]. Risk factors for CRC include those stipulated for cancer in general, and they are exacerbated by obesity, diabetes mellitus, insulin resistance, acromegaly, and immunosuppression [40]. Non-modifiable risk factors include age, gender and genetic factors, such as the family history of CRC or adenomatous colonic polyps, long-standing inflammatory bowel disease, familial adenomatous polyposis, and hereditary non-polyposis CRC. Its prevention is primarily through colon cancer screening and avoiding modifiable risk factors [41,42]. People at risk or suffering from CRC present with a combination of symptoms, which include rectal bleeding, abdominal pain, intestinal obstruction or perforation, constipation, unexplained weight loss and iron deficiency anemia [41].

## 3. Conventional Treatment and Management of Cancer

Conventional cancer therapies include surgery, radiotherapy, chemotherapy and immunotherapy, depending on the type of cancer, stage of the cancer and cancer site. The primary treatment of cancer is usually surgery or radiotherapy, while chemotherapy or immunotherapy may be used before or after the definitive treatment as “adjuvant” or “neo-adjuvant” therapy, respectively [43,44]. Surgery involves the excision of tumors; it is used for reduction in the tumor size (termed debulking) or the complete removal of organs to prevent the further spread of the cancer to other organs [45]. Surgery improves quality of life and increases the survival rate of cancer patients. However, it is costly, invasive, and most effective for benign tumors. Additionally, tumor relapse can occur following surgery, particularly when the tumor has metastasized [46].

Radiotherapy employs X-rays, gamma rays and neutrons that damage the DNA of cancer cells. It is non-invasive, cost-effective and suitable for localized tumors. Also, it improves the cure rate when used in combination with surgery (pre- or post-operative) and/or chemotherapy [47]. Radiotherapy also relieves pain in advanced or metastatic cancer. However, the substantial capital required for facilities and equipment limits access, particularly in low- and middle-income countries [47]. Although radiation is more harmful to cancer cells than normal cells, it does not discriminate between cancer and healthy cells, leading to various side effects such as hair loss, nausea, vomiting, immunosuppression and myelosuppression due to its effects on gastrointestinal cells, skin, hair and other functional organs [43,48]. Radiation could cure or cause cancer owing to its lack of specificity. Following radiation, the sudden disintegration of cancer cells may lead to the release of cancerous cells into lymphatic nodes, thus forming tumors in healthy organs [49].

Immunotherapies such as bevacizumab, cetuximab, panitumumab, and ramucirumab [4] stimulate the immune system to recognize and attack cancer cells. This approach complements or stimulates the immune system through lymphokines, cytokines, vaccines, tumor-specific antibodies or effector cells [50]. This therapy prevents the metastasis of cancer and has been reported to prolong the overall survival rate of colorectal, breast, bladder and cervical cancer [51]. Nonetheless, immunotherapy presents challenges such as high treatment cost, autoimmune reactions to therapeutics, high immune-mediated toxicity, unpredictable efficacy and the development of resistance attributed to increased hypoxia, acidity and abnormal neovascularization within the tumor microenvironment [52,53].

Chemotherapy uses cytotoxic agents to destroy malignant cells. This strategy destroys cancer cells in early stages, slows down tumor metastasis and can be used alone or in combination with radiotherapy and surgery to shrink tumors in a neo-adjuvant or adjuvant approach [30]. Chemotherapy increases survival rates in several cancer types, including colorectal, breast, prostate, Hodgkin’s lymphoma and pediatric acute lymphoblastic leukemia, among others [54]. Anticancer drugs used in CRC treatment include dox, docetaxel, leucovorin, 5-FU, and irinotecan [4]. These drugs have not been effective in increasing the overall survival of CRC patients, particularly in metastatic cancer, due to the inability of chemotherapeutics to penetrate the tumor microenvironment and their associated side effects. Generally, chemotherapeutic drugs have many drawbacks that limit their application primarily due to their non-selectivity and associated adverse effects on healthy cells. Their non-selectivity destroys both cancer and normal cells, particularly rapidly dividing cells, such as bone marrow cells, digestive tract cells, and hair follicles, leading to myelosuppression, mucositis, alopecia or immunosuppression [45,55]. Cancer chemotherapeutics also have poor aqueous solubility and a short half-life [46]. Another challenge in cancer chemotherapy is the development of multidrug resistance, which occurs through various mechanisms, such as reduced drug uptake due to the overexpression of P-glycoprotein, drug efflux out of the tumor due to hydrostatic pressure in the tumor interstitium, and neo-vasculature [56]. Consequently, these challenges have led to an exigent need for newer approaches for effective drug delivery for cancer treatment. Considering these challenges, the goal of cancer therapy has shifted toward targeted drug delivery, which facilitates drug uptake into diseased cells while preventing bystander effects on non-cancer cells. Hence, aptamers combined with AuNPs are perceived as candidates for developing biocompatible targeted drug delivery directed to CRC-tumor sites.

## 4. Nanotechnology-Based Strategies in Cancer Therapy

**Figure 1:**
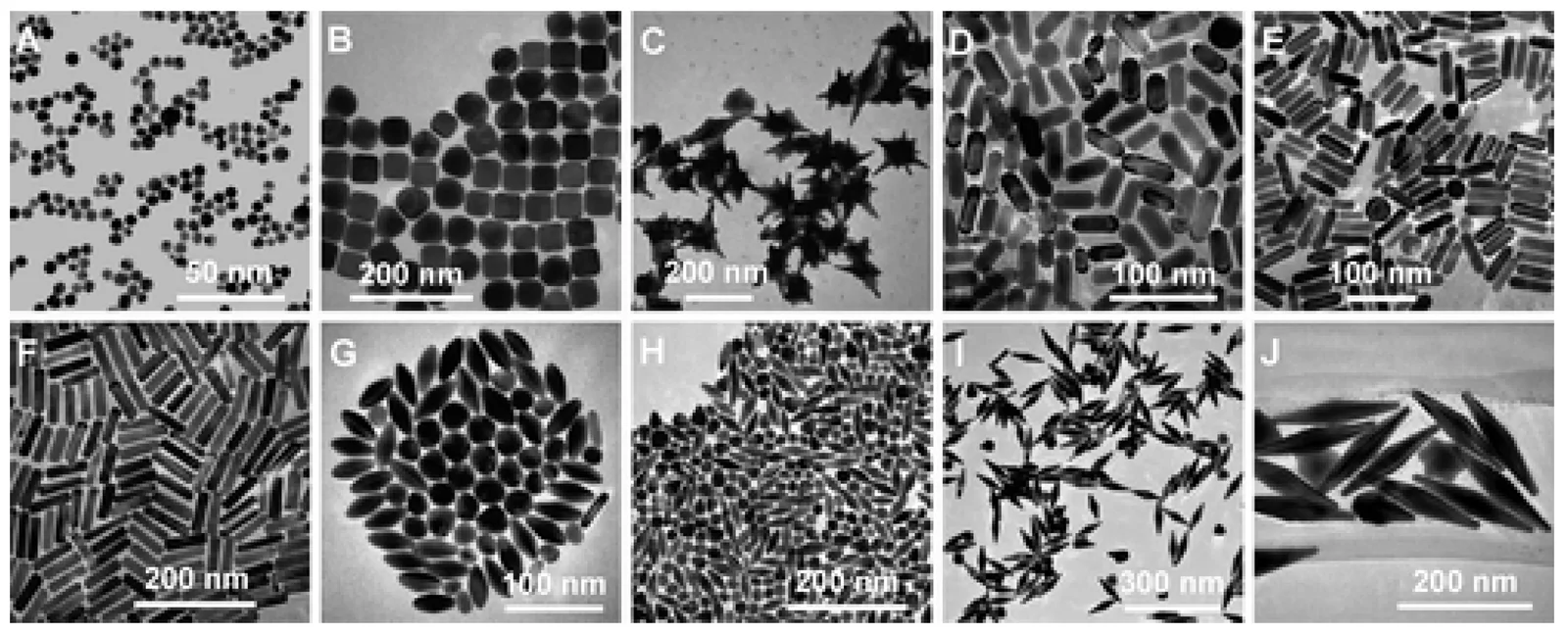
Sizes and shapes of AuNPs captured through transmission electron microscope. Shapes include Au (**A**) nanospheres, (**B**) nanocubes. (**C**) nanobranches. (**D**–**F**) nanorods, (**G**–**J**) Nanobipyramids. Reprinted with permission from Langmuir [64].

Nanotechnology is a multi-disciplinary field that involves the fabrication of nanomaterials in the 1–100 nm dimension for various applications [57,58]. Their unique physico-chemical properties, such as the small size, surface reactivity and shape, that are shown in Figure 1, are the highlights of their application in biomedicine as therapeutic, drug delivery, imaging, labeling, sensing and diagnostic agents [9]. These characteristics ensure targeted drug delivery to specific cells and tissues while reducing harmful effects on normal cells. Consequently, nanomaterials increase drug efficacy, lower drug toxicity, and prolong drug circulation time [59]. These properties have been explored in the diagnosis and treatment of several diseases such as cancer [60], microbial (fungal and bacterial) infections [61], obesity [62], and diabetic nephropathy [63], among others.

In cancer therapy, various nanomedicines that explore the exceptional properties of organic (polymer, micelle, protein, liposome) and inorganic (gold, silver, titanium and calcium) nanomaterials have received United States Food and Drug Agency (US FDA) approval, while some are currently undergoing clinical trials. Organic NPs were the first US FDA clinically approved nanocarriers. For example, Doxil, dox-loaded liposomes, was approved in 1995 for the treatment of ovarian cancer, metastatic breast cancer, multiple myeloma, CRC and Karposi’s sarcoma associated with Acquired Immune Deficiency Syndrome [65]. The formulation reduced cardiotoxicity, gastrointestinal toxicity, alopecia and extravasation necrosis in patients [66,67]. In 2015, Onivyde, liposomes loaded with irinotecan, was approved for the treatment of advanced pancreatic adenocarcinoma. It had prolonged circulation time, sustained drug release, and reduced off-target effects in treated patients [68]. Abraxane, an albumin-bound paclitaxel, was clinically approved for the treatment of pancreatic, breast, and non-small-cell lung cancers. It also improved drug efficacy in the treatment of metastatic breast cancer, reduced anemia and neutropenia in treated patients [69,70]. Several nanomedicines are investigated in clinical trials for various cancer types, including CRC (Table 1).

**Figure 2:**
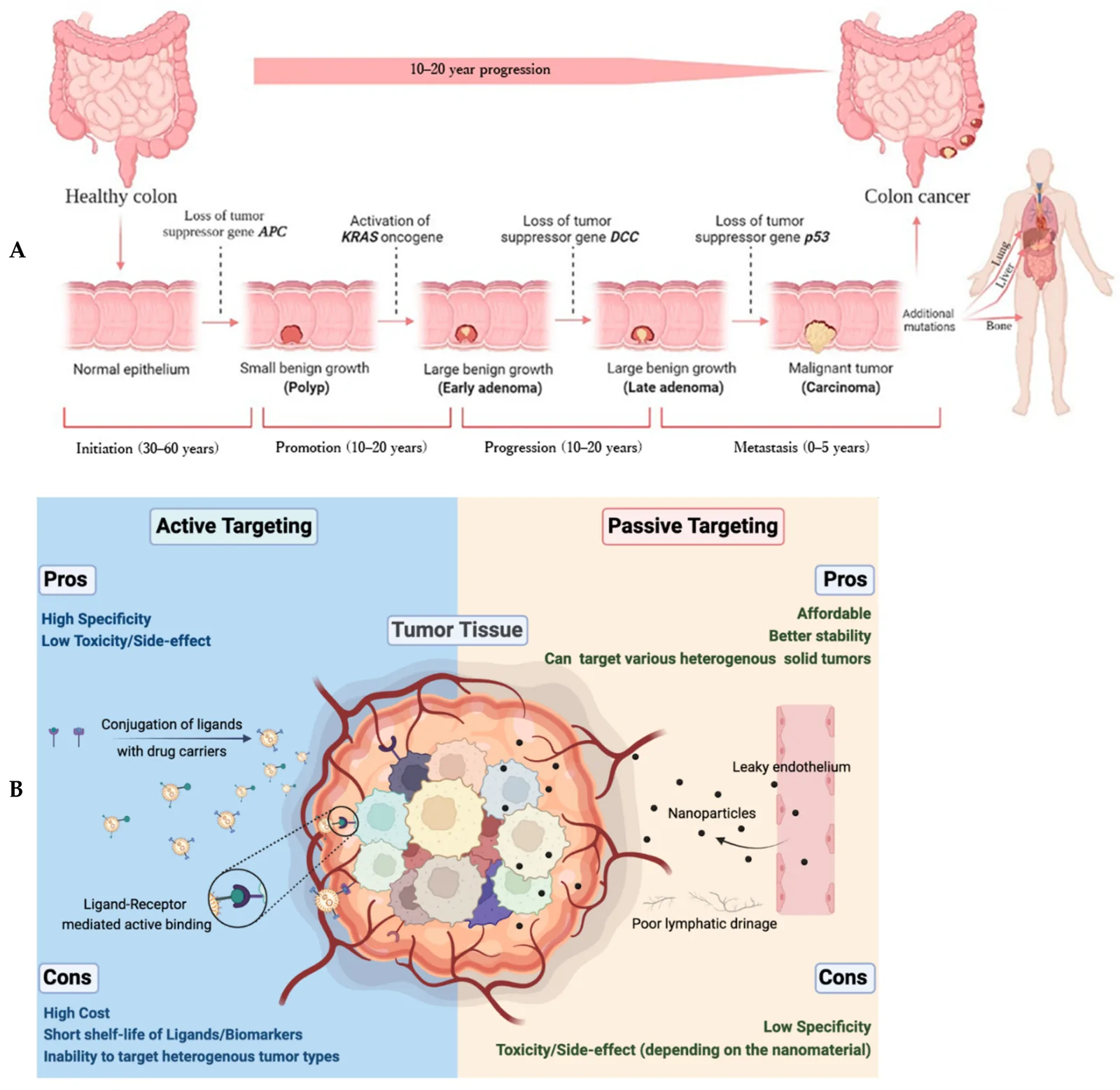
Stages of CRC and the exploration of disease-specific biomarkers for targeted treatment strategies. (**A**) Development and progression of CRC and (**B**) role of EPR effect in NP-based treatment strategies. Active targeting exploits biomarkers that are expressed in each stage of CRC for cell-specific targeting. The defective vasculature and impaired lymphatic drainage of tumors allow the preferential accumulation and retention of NPs within cancer cells through passive targeting. (**A**) was reprinted from MDPI [73]. (**B**) Created in BioRender (https://www.biorender.com, accessed on 15 July 2025).

These nanomedicines rely on the size of NPs and the enhanced permeability and retention (EPR) effect in diseased tissues for targeted treatment. It is well established that the vasculature of solid tumors is characterized by hypervascularization, defective vascular architecture and reduced lymphatic drainage [60], presenting a gateway for passive drug uptake and delivery. Consequently, nanomedicines can preferentially penetrate tumor cells using the EPR effect to target diseased cells and are retained for a longer period at the target site due to impaired lymphatic drainage. The physicochemical properties of NPs, particularly their size, can be tailored to influence their biodistribution, cellular uptake, circulation time, and tumor retention. Size manipulation is essential because smaller NPs are rapidly excreted by RES organs such as the kidney, liver and spleen, whereas larger NPs are extravasated from cellular entry and phagocytosed by the RES [71]. Active targeting uses molecules that recognize disease-specific biomarkers to enhance treatment specificity. As illustrated in Figure 2A, CRC can progress from Stage I to IV with each stage characterized by the differential expression of specific biomarkers [72]. Targeting these biomarkers may therefore improve the selective delivery of nanomedicines to CRC.

The size of nanomaterials, with emphasis on MNPs, can be harnessed for the passive and active targeting of cells (Figure 2B). In passive targeting, it is widely established that tumor cells have defective vasculature; hence, nanomaterials can passively traverse into diseased cells through the EPR effect [74]. On the other hand, active targeting leverages the fact that cancer cells exclusively overexpress certain cellular receptors. Hence, biologically relevant molecular recognition elements such as aptamers, antibodies, and peptides that target these receptors can be conjugated onto the NPs so that they can bind preferentially to cancer cells, ultimately increasing their bioavailability in the desired region [75]. MNPs, with a special focus on AuNPs, have additional benefits compared to organic NPs, which are primarily used as drug carriers. The metal core is responsible for the multiple functions of the MNPs, which can be controlled by varying their sizes and shapes during synthesis. It is widely reported that spherical AuNPs that are ≤50 nm localize into internal organs, such as the heart, spleen, liver and kidney, as well as deep layers of the skin, while AuNPs at 100–200 nm largely remain on the cell surface and are not internalized across biological barriers such as endothelial junctions, extracellular matrix in tumors, and the blood–brain barrier [29]. Based on this observation, AuNPs have received a lot of interest in biomedicine as diagnostic and therapeutic agents. Smaller AuNPs (10–15 nm) have been utilized for drug delivery, 30 nm Au nanospheres (AuNSs) for diagnosis, while 50 nm AuNSs conjugated with antibodies demonstrated theranostic potential [76].

### 4.1. Properties of AuNPs as a Promising Tool in Cancer Therapy

AuNPs have been used in many fields such as biotechnology, pharmacy, material science and engineering [77]. They have attracted considerable interest due to their shape and size-dependent properties, including chemical stability, tunable optical activity, photothermal conversion ability, low cytotoxicity and, most importantly, a large surface-area-to-volume ratio [78,79]. AuNPs of different sizes and shapes can be synthesized using either “top–down” or “bottom–up” approaches. The top–down approach breaks down bulk material into nano-sized structures [8,80]. This approach requires a substantial amount of energy to maintain high temperature and pressure conditions for synthesis [80,81]. Top–down methods include laser ablation, ion sputtering, ultraviolet and infrared irradiation, and aerosol technology [82]. In contrast, the bottom–up approach assembles small atoms or reduces of ions to form desired nanostructures. This approach follows chemical reduction or biological methods; it is preferably used because it is easy to control the size distribution and shape of NPs and requires a lower amount of energy [83,84]. The chemical method employs chemicals as reducing and stabilizing agents. The reducing agents include borohydride, oxalic acid, sodium citrate, and citric acid, while stabilizing agents include thiol-containing molecules, polymers and surfactants. Examples of chemical methods include the Turkevich, seeding, Brust–Schriffin, and electrochemical methods [85]. Biological synthesis uses microbes, algae, fungi and plants, which are environmentally friendly as reducing and capping agents [85,86]. The size and shape of the AuNPs define their downstream applications. Various shapes such as nanorods, nanospheres, nanostars, nanocubes, and nanocages [87] have been used as drug delivery, photothermal, labeling, and sensing agents for disease diagnosis and treatment. Owing to these properties, AuNPs have also been widely explored in the treatment of cancer and other diseases [88], as described elsewhere [74].

#### 4.1.1. AuNPs as Drug Delivery Agents

AuNPs have good surface properties. They have a large surface-area-to-volume ratio that allows for the loading and conjugation of biomolecules of interest. Because AuNPs have a highly reactive surface, they are easy to functionalize with various biomolecules to enhance their stability, solubility and pharmacokinetic properties [89]. AuNPs have been successfully used as drug delivery agents in the treatment of various diseases. This has been achieved by conjugating AuNPs with therapeutic agents such as antibiotics and anticancer drugs as well as targeting peptides such as antibodies and aptamers.

In CRC therapy, AuNPs are promising tools for the delivery of chemotherapeutics to CRC cells. Table 2 summarizes the drug delivery and therapeutic effects of AuNPs on various CRC cells. AuNPs have efficiently transported and delivered anticancer agents to CRC cells; these include chemotherapeutic drugs such as cetuximab [21], dox, cisplatin, paclitaxel, methotrexate [20], irinotecan, and bleomycin as well as plant-derived compounds (quercetin [90], curcumin [91]). For instance, AuNPs functionalized with cetuximab showed higher cytotoxicity against colon cancer (HT-29) cells when compared to free cetuximab. In the study, citrate-capped AuNPs were conjugated with cetuximab; cell viability and apoptosis assays were then performed. The results revealed that the cetuximab-AuNPs reduced cell viability by more than 60%, while the free AuNPs decreased cell viability by 19.1%. Cells treated with cetuximab-AuNPs died through apoptosis at 27.86% compared to 15.68% and 12.22% obtained from free cetuximab and unconjugated AuNPs, respectively [21]. AuNPs have also been used to enhance the anticancer efficacy and reduce the side effects of 5-FU against CRC cells. In this experiment, 5-FU was loaded onto AuNPs using thiol-containing ligands, thioglycolic acid, and glutathione (GSH), producing 5-FU/GSH-AuNPs. CRC cells were incubated with 5-FU-loaded and unloaded AuNPs. The study revealed that 5-FU from AuNPs was slow-released and induced apoptosis [92]. In another study, Lee et. al. [93] exposed SW480 colon cancer cells to dox-loaded AuNPs coated with oligonucleotides; three groups of mice were further administered free dox, dox-loaded AuNPs and PBS. The results indicated that cells treated with 10 µM dox-loaded AuNPs showed a 42% decrease in cell viability while free dox showed negligible cell death. Likewise, dox-loaded AuNPs and free dox showed a 30% and 10% decrease in tumor volume, respectively, when compared to PBS-treated control, demonstrating that AuNPs enhance the cytotoxic potential of dox [93]. Liszbinski and colleagues also demonstrated that 5-FU-conjugated AuNPs exert cytotoxic effects in human CRC (HCT 116 and HT-29) cells, where they decreased cell proliferation through apoptosis and necrosis [94]. AuNPs have also been used to deliver phytochemicals to CRC cells; the conjugation of quercetin to AuNPs significantly enhanced cytotoxicity in SW620 cells [95]. Curcumin conjugated to AuNPs displayed significant cytotoxicity and cellular uptake in HT-29 cells [90]. The studies presented in Table 2 indicate that AuNPs are excellent vehicles for the delivery of conventional drugs (cetuximab, 5-FU, methotrexate, dox, cisplatin), as they improved drug efficacy in the treatment of various CRC cells.

AuNPs-based formulations have been approved by the FDA for human clinical trials for disease treatment; among those registered in ClinicalTrials.gov is AuroLase (NCT01679470), which is composed of gold-silica nanoshells for the treatment of prostate cancer. It is used in photothermal therapy to induce hyperthermia and coagulative necrosis [14]. CYT-6091 (NCT00356980), a PEGylated 27 nm AuNP functionalized with tumor necrosis factor (TNF), is in phase 1 clinical testing and has shown improved therapeutic outcomes in advanced solid tumor in patients [99]. Silica-AuNPs (NCT01270139) are in a phase 1 trial for the management of coronary atherosclerosis. Likewise, AuNPs are in phase 1 clinical trials as delivery vehicles for vaccines against Severe Acute Respiratory Syndrome Coronavirus 2 (naNO-COVID-19, NCT05113862) and dengue fever EMX-001 (NCT04935801) [14].

#### 4.1.2. AuNPs as Photothermal Agents

AuNPs have exceptional optical properties, which are attributed to their SPR. Depending on their size, shape and optical properties, AuNPs can absorb light from the visible to near-infrared region (520–800 nm). SPR changes when light strikes the AuNPs, causing electron oscillation. These oscillations emit photons and induce heat, and they can be used in photothermal and photodynamic therapy [100]. This unique SPR property is shape-dependent and common to gold nanorods, nanoshells, nanostars, and nanocages, as highlighted by the studies summarized in Table 3. Goodrich and colleagues reported increased survival time in murine colon cancer xenograft mice exposed to laser only, gold nanorods, and gold nanorods plus laser irradiation. After 24 h treatment, the mean survival time for the photothermal ablation group, nanorods only, ‘laser only’ was 42.1, 9.7 and 9.1 days, respectively, while control groups only survived for 8 days. It was also observed that 44% of the ‘nanorod plus laser’-treated mice survived and showed complete tumor ablation after 60 days [101].

In another study, SW-620 colon cancer cells were incubated with branched gold nanoshells for 3 h and irradiated with a laser at 800 nm for 10 mins. After this, the cells were treated with 76 nM 5-FU for 24 and 48 h, which was followed by a cell viability assay. Also, SW-620 cells were injected into Nunu murine mouse models and treated with 5-FU, branched gold nanoshells and laser irradiation. The results revealed that AuNPs in combination with 5-FU caused a marked reduction in cell viability and tumor growth compared to the untreated control [102]. Gold nanoshells (Auroshells) are the most advanced in cancer photothermal therapy and are currently in clinical trials for treating head, neck, lung, and prostate cancer. They are reported to have a good safety profile, but studies regarding their efficacy are still ongoing and have not been published [103].

**Table 3:** Photothermal properties of AuNPs in CRC cells.

AuNPs in Photothermal Therapy	Cancer Model	Outcome	Reference
PEGylated gold nanorods	Murine colon cancer (CT-26) model	Significantly increased survival time in nanorod plus laser-exposed mice and tumor ablation in BALB/c mice.	[101]
Gold nanoshells administered with 5-FU	Murine colon cancer (SW-620) model	Decreased cell viability and inhibited tumor growth in mice.	[102]
Platinum (II)-loaded gold nanoshell	Human CRC (HT29 and LS174T) cells	Photothermal therapy caused profound decrease in cell viability of cancer cells and tumor suppression with no toxic effect in BALB/c nude mice.	[104]
Enzyme-conjugated gold nanostars	Colon cancer (SW-480) cells	Induced cytotoxicity and increased apoptosis in irradiated cells.	[105]
Vertepofin-loaded gold nanorods	Human colon cancer (HCT 116) cells	Decreased cell viability after laser irradiation and inhibited tumor growth in cancer xenograft.	[106]
Gold nanostars	Colorectal adenocarcinoma (DLD-1) cells	NIR irradiation induced cytotoxicity and necrosis in CRC cells.	[107]
Anti-EGFR gold nanorods loaded with methotrexate	Human CRC (HCT 116 and HT-29) cells	Cell death increased in cells irradiated with conjugated gold nanorods compared to unconjugated gold nanorods.	[108]
Dox loaded gold nanocage	Murine hepatocellular carcinoma (H22) cells	Significantly decreased cell viability and reduced tumor growth in mice.	[109]
Gold nanosphere	Colorectal adenocarcinoma (DLD-1) cells	Markedly enhanced apoptosis and necrosis in CRC cells.	[110]
PEGylated gold nanorods	Squamous cell carcinoma xenograft	NIR irradiation caused significant decrease in tumor size and tumor resorption in nu/nu mice.	[111]

#### 4.1.3. AuNPs in Disease Therapy

AuNPs, among other MNPs, have gained interest in therapeutics due to their perceived low cytotoxicity. Their biocompatibility is attributed to the non-toxicity of their bulk material and its clinical use in disease treatment, including cancer [112]. Studies have shown that the effects of AuNPs are size- and dose-dependent with smaller sizes and higher doses showing higher cytotoxicity [113,114]. A preclinical study demonstrated dose-dependent effects of 12.5 nm AuNPs in mice administered doses of 40, 200, and 400 µg/kg/day. Histological analysis revealed that the AuNPs accumulated in several organs, including the liver, spleen, kidneys, and lungs [115]. Another study reported that the AuNP size significantly influences their biodistribution in rats. Higher levels of 7 nm AuNPs accumulated in the lungs, esophagus, kidneys, and brain compared with 20 nm AuNPs [116]. These studies indicated that smaller AuNPs may exhibit greater tissue distribution [115,116]. Lopez Chavez et al. further investigated the effects of AuNP size using 10, 30, and 60 nm citrate-capped AuNPs in human CRC (HT-29) cells, hepatocellular carcinoma (HepG2) cells, and Wistar rats. The 10 nm AuNPs exhibited greater tissue biodistribution, whereas the 30 and 60 nm AuNPs were detected predominantly in the liver, intestine, kidneys, spleen, urine, and feces of the rats. In addition, the 10 and 30 nm AuNPs exhibited greater cytotoxicity than the 60 nm AuNPs with exposure associated with increased DNA damage in the cell nucleus and elevated reactive oxygen species (ROS) production [117]. 

The drug-sensitizing effects of AuNPs synthesized from *Cyclopia intermedia* and *Mangifera indica* were revealed in a study by Aboyewa et al. Colon cancer (Caco-2) cells were co-treated with non-lethal concentrations of dox and the AuNPs; the AuNPs significantly enhanced the drug’s cytotoxicity on the cells. The co-treatment with AuNPs from *Cyclopia intermedia* and *Mangifera indica* reduced Caco-2 cell viability by 70% and 40%, respectively [25]. These studies showed that AuNPs have anticancer potential and could be used to reduce the bystander effects of conventional chemotherapeutics. However, their small size could result in non-specificity and allow the internalization of AuNPs into off-target organs through passive targeting [26]. To overcome this limitation, AuNPs can be conjugated with relevant targeting moieties such as antibodies, targeting peptides, and aptamers to confer selectivity as well as increase their translocation into the target site.

To ensure targeted delivery, AuNPs have been surface-modified with cetuximab, which is an EGFR-specific antibody. In the study, gold nanorods conjugated with cetuximab caused higher absorption and cellular damage in EGFR-expressing cancer cells compared to non-EGFR-expressing cells. Further studies in vivo revealed that when pancreatic cancer xenografts mice were treated with the AuNPs–cetuximab conjugate followed by laser irradiation, a significant decrease in tumor size, increased necrosis and higher caspase-3 activity were observed [21]. In another experiment, gold/silica nanoshells were conjugated with antibodies specific for interleukin 13Rα2 that is highly expressed in glioma (U373 and U87) cells. AuNPs–antibody conjugates induced cell death in glioma cells but not in A431 epidermoid carcinoma cells due to low expression of the IL-13 receptor [118]. These studies indicate the potential of using targeting moieties as tools for targeted drug delivery [40,119]. 

## 5. Aptamers in Targeted Treatment Strategies for CRC

Antibodies play a central role in targeted cancer therapy by recognizing specific molecular signatures (biomarkers) associated with cancer development and progression, thereby facilitating the selective inhibition or destruction of cancer cells. In CRC, antibody-based therapies include cetuximab and panitumumab, which target the EGFR, and bevacizumab, which targets VEGF [120]. Despite their therapeutic benefits, antibody-based therapies have several limitations, including high production costs, potential immunogenicity, and the need for antibody humanization to minimize adverse immune responses [121,122]. Consequently, aptamers, also called chemical rivals of antibodies, are now taking precedence over antibodies owing to several advantages. They have higher affinity, specificity, and thermal stability, and they could serve as alternatives to antibodies. They are cost-effective, easy to synthesize, have low immunogenicity and are stable at various temperatures as well as pH conditions [24,123,124]. Similarly, they can be readily modified by the attachment of linkers, drugs, analytes and other biologically active molecules. Based on these unique features, aptamers have been used in disease treatment, including cancer [123]. Their increasing relevance in disease treatment is due to their ability to target various biological molecules, including non-immunogenic targets [122,123].

Aptamers are short, single-stranded DNA or RNA oligonucleotide that have high affinity and specificity for their target molecules, such as proteins or cell receptors. They are very small in size (between 20 and 60 nucleotides), and they have low molecular weights between 3 and 5 nm in diameter [125]. In 1990, Tuerk and Gold reported the first aptamer against bacteriophage T_4_ DNA polymerase [126]. Ellington and Szostak also produced RNA ligands against small molecule dyes using Systematic Evolution of Ligands by EXponential Enrichment (SELEX) [127]. The SELEX technique is an in vitro and scalable method for identifying high-affinity aptamers. It involves screening a large library of oligonucleotides against a specific target through multiple iterative selection rounds, typically ranging from 5 to 10 cycles or more depending on the target and selection conditions. During each cycle, target-bound nucleic acids are separated from unbound sequences, and the selected sequences are subsequently amplified by polymerase chain reaction (PCR) to generate an enriched library for the next round of selection [126,128,129]. 

Several modifications have been developed to overcome the limitations of conventional SELEX and improve the specificity and efficiency of aptamer selection. These include cell-SELEX, counter-SELEX, and exonuclease-based digestion of small extracellular vesicles (EDGE)-SELEX, which have been employed for identifying aptamers targeting CRC. Cell-SELEX involves the screening of RNA or DNA libraries directly against intact, living CRC cells, including Caco-2, HCT-116, and DLD-1 cells, allowing aptamers to be selected against cell-surface molecules [130]. In counter-SELEX, the aptamer library is first exposed to non-malignant colorectal cells to eliminate sequences that bind non-specifically to normal cells. The unbound sequences are subsequently subjected to positive selection against CRC cells, thereby enriching aptamers that preferentially bind to cancer cells [131]. In contrast, EDGE-SELEX selects aptamers against small extracellular vesicles derived from CRC cells as targets. The unbound DNA in solution is digested by exonucleases, thereby eliminating the step for physical immobilization of the extracellular vesicles [132].

Despite these advances, SELEX-based approaches generally require multiple iterative selection and amplification cycles, making them relatively time consuming and potentially associated with sequence bias and limited selection efficiency. Consequently, in silico approaches have emerged as complementary strategies for aptamer discovery. These computer-aided methods employ techniques such as molecular docking and molecular dynamics simulations to predict and evaluate interactions between aptamers and their targets. Compared with conventional SELEX, in silico selection can reduce experimental requirements and costs while also accelerating the identification of candidate aptamers [133].

### 5.1. Advantages of Aptamers over Antibodies

Aptamers possess several properties comparable to those of antibodies, but they also offer several advantages that are superior to antibodies. They are relatively easy to synthesize outside a biological system through SELEX compared to the in vivo or in vitro processes required for antibody production and purification [134]. The aptamer selection processes overcome the batch-to-batch variation encountered with antibody production [135]. They are also easy to modify by conjugation with drugs, fluorophores, quantum dots, reporters and linkers [136] with no significant effect on their binding affinity and activity. This property also ensures that the pharmacokinetic parameters of aptamers can be manipulated compared to antibodies whose pharmacokinetics are difficult to modify [137].

Furthermore, aptamers are very stable and remain intact in temperatures ≥ 95 °C, varying pH conditions (4–9), and in the presence of organic solvents, whereas antibodies are permanently denatured when exposed to heat and unfavorable pH conditions [138,139]. Aptamers have a highly ordered structure that forms stable complexes with biological components such as proteins, nucleic acids, drugs and other small molecules [136,140]. Aptamers are biocompatible and considered useful alternatives to antibodies as they exhibit little to no toxicity or immunogenicity, while antibodies could elicit significant levels of immune responses [141]. More importantly, aptamers have very high affinity for their targets; they show dissociation constants that range from micromolar to femtomolar. Owing to this, they possess the ability to discriminate between target proteins that share structural similarities [137,138]. Consequently, aptamers have gained prominence as potential tools for diagnostic and therapeutic applications.

### 5.2. Application of Aptamers in Therapeutics

Aptamers have diverse applications in biomedicine, where they serve as targeting, delivery, and therapeutic moieties. As targeting ligands, aptamers can selectively direct therapeutic cargoes to specific cells or tissues, thereby enhancing targeting specificity. Owing to their high affinity and specificity for a broad range of biological targets, aptamers can also be employed for the detection of disease-associated biomarkers in diagnostic applications and for the targeted delivery of therapeutic agents. As therapeutic agents, aptamers can modulate disease-related processes by interfering with protein–protein, nucleic acid–protein, or receptor–ligand interactions, thereby inhibiting the activity of disease-associated proteins [142].

Pegaptanib (Macugen) produced by Pfizer Inc. (New York, NY, USA) was the first aptamer approved by the US FDA in 2004. The 28 nt RNA aptamer was selected after 10 rounds of cell SELEX, and it was developed for age-related macular degeneration (AMD) and other solid tumors. The anti-angiogenic agent inhibits VEGF, which is a receptor that is essential for the formation of tumor blood vessels [143]. Subsequently, other aptamers are undergoing clinical trials for other diseases. For example, EYE 001 (NCT00056199), an anti-VEGF aptamer, has reached phase 2 and 3 clinical trials for the treatment of AMD [144]. NOX-E36 (NCT00976729), a L-RNA aptamer, is in a phase 1 clinical trial for the treatment of diabetic nephropathy, while REG1 (NCT00715455), an aptamer that is highly specific for coagulation factor IXa is in a phase 1 clinical trial for treating coronary artery disease [145,146].

Aptamers are currently being explored for treatment of various cancer types. For example, CD44- and Epithelial Cell Adhesion Molecule (EpCAM)-specific RNA aptamers have demonstrated the ability to induce apoptosis and reduce cell proliferation in ovarian cancer cells. Moreover, the bispecific aptamer suppressed tumor growth in an ovarian cancer model [147]. Olaptesed, NOX-A12 (NCT03168139), is a PEGylated mirror-image RNA aptamer with high specificity for chemokine CXCL12 implicated in angiogenesis, cell proliferation, and the metastasis of leukemia. Olaptesed pegol has advanced to phase II clinical trials for the treatment of chronic lymphocytic leukemia, multiple myeloma, metastatic CRC and pancreatic cancers [148]. The osteopontin (OPN)-R3 aptamer binds OPN, which is a phosphoprotein implicated in tumorigenesis, tumor growth, and metastasis across several types of cancer. OPN-R3 aptamer treatment inhibited cell adhesion, migration, and invasion in MDA-MB-231 breast cancer cells [149]. Taken together, these studies further demonstrate the therapeutic potential of aptamers in cancer management. 

Several other studies have shown aptamer-based systems as promising candidates for drug delivery and therapeutics. In their targeting roles, aptamers have been conjugated with anticancer drugs such as docetaxel [150], dox [151] and gemcitabine [145] to enhance drug biodistribution and efficacy. Trinh et al. demonstrated that a DNA aptamer (AS1411) conjugated to dox inhibited tumor growth and selectively targeted hepatic carcinoma in a mice xenograft [151,152]. AS1411 also displayed high tumor suppression when combined with gemcitabine in pancreatic and lung cancer xenograft models [145,153]. Likewise, E3 aptamer, which is highly selective for prostate cancer antigen, was conjugated with mono-methyl auristatin E and methyl auristatin. The conjugates elicited significant in vivo cytotoxic activity against prostate cancer cells but not normal cells [154,155]. PSMA-specific aptamers conjugated with dox improved drug efficacy in PC-3 cells and suppressed the growth of PSMA-positive antigens [26]. Treatment with an aptamer/microRNA-inhibitor complex also improved the delivery of therapeutic agent and reduced tumor growth in an orthotopic ovarian cancer mouse model [145], further demonstrating the potential application of aptamers in cancer therapy.

#### 5.2.1. Aptamer-Targeted Strategies in CRC

Aptamers targeting molecular markers associated with CRC can function as either targeting moieties or antagonists. As targeting ligands, aptamers can selectively deliver therapeutic cargoes to CRC cells, whereas antagonistic aptamers can inhibit specific receptors or molecular pathways involved in CRC development and progression. By enhancing target specificity, these approaches could reduce limitations associated with conventional CRC therapies and potentially minimize unintended damage to healthy cells. Several aptamers have been developed against molecular markers implicated in CRC pathogenesis and progression; these are summarized in Table 4.

MUC-1 is a cell surface glycoprotein that is highly expressed in CRC, breast, prostate and pancreatic cancer [156]. MUC-1 aptamer, 5TR1, conjugated with hyaluronan/chitosan NPs was used for the efficient delivery of 5-FU. The exposure of human CRC adenocarcinoma and Chinese hamster ovary (CHO) cells to the MUC-1 aptamer conjugate resulted in an increased cytotoxic effect in CRC adenocarcinoma cells compared to MUC-1 negative CHO cells [157].

The SYL3C aptamer that targets EpCAM in CRC was investigated for CRC treatment [158]. The SYL3C aptamer was conjugated to dox-loaded liposomes and used for the efficient delivery of dox to C26 CRC cells and CHO cells. The treatment resulted in an increased cellular uptake of dox into C26 cells compared to CHO cells with an enhanced tumor accumulation of dox and increased survival in female BALB/c mice [159]. 

Carcinoembryonic antigen (CEA) is another biomarker that is expressed in CRC and is closely associated with cell adhesion, anoikis resistance and liver metastasis [160]. CEA aptamer co-treatment with 5-FU was tested against 5-FU-sensitive and chemo-resistant LS174T CRC cells. Treatment with the combination of CEA aptamer and 5-FU sensitized the chemo-resistant cells to more than 5-fold (IC_50_~5.995 μM) compared to cells treated with 5-FU alone (IC_50_~31.46 μM). The CEA aptamer further regressed tumor volume in a male BALB/cAnNCrI xenograft model [161]. 

AptS100P-1, a DNA aptamer targeting S100P, a protein involved in the regulation of cell growth, invasion, and metastasis, has been investigated as an antagonist aptamer in DLD-1 human CRC cells. The treatment of S100P-expressing DLD-1 cells with AptS100P-1 reduced cell proliferation and migration. Moreover, in vivo studies demonstrated that AptS100P-1 inhibited tumor growth in BALB/c nude mice, highlighting its potential as a therapeutic antagonist for CRC [162].

**Table 4:** Aptamers, target biomarkers and their outcomes in CRC treatment.

CRC-Related Aptamers	Type of Biomarker	Outcome	References
5TR1	MUC-1	Increased uptake of 5-FU into CRC adenocarcinoma compared to MUC-1 negative control	[157]
SYL3C	EpCAM	Increased cellular uptake of dox into C26 CRC cells, enhanced survival in female BALB/c mice	[159]
CEA aptamer	CEA	Sensitized chemo-resistant LS174T CRC cells to 5-FU and regressed tumor volume in BALB/cAnNcRI xenograft model	[161]
AS1411	Nucleolin	Decreased cell viability in SW-480 cells in a dose-dependent manner while causing no effect on normal colon cells	[163]
EpCAM aptamer (Apt)	EpCAM	Delivered miRNA to EpCAM positive cells, inhibited cell growth and suppressed tumor growth in HCT-8 tumor-laden mice	[164]
Apt S100P-1	S100P	Decreased migration and growth of DLD-1 CRC cells, inhibited tumor growth in BALB/c mice	[162]
L33	Circulating tumor cell	Caused selective internalization of dox into HCT-116 CRC cells, elicited cytotoxicity in HCT-116 cells compared to non-target CL187 cells	[165]

#### 5.2.2. Aptamer-Targeted AuNPs-Based Strategies for CRC

Targeted therapy represents a promising strategy for overcoming the limitations of conventional CRC treatment. Several molecular features and signaling pathways associated with CRC development and progression have been identified and provide potential targets for improving therapeutic outcomes [71]. In this approach, aptamers can function as targeting ligands to selectively direct therapeutic agents to CRC cells, thereby enhancing target specificity and reducing off-target toxicity. Meanwhile, AuNPs can serve as nanocarriers for the delivery of therapeutic payloads. Therefore, the combination of aptamers and AuNPs provides a multifunctional platform that integrates active targeting with passive tumor accumulation through the EPR effect. This combination could generate synergistic effects by integrating targeting, drug delivery, and therapeutic functions within a single nanosystem. 

In CRC treatment, aptamer-conjugated AuNPs have shown considerable potential for targeted drug delivery by promoting tumor accumulation and reducing the nonspecific effects associated with conventional chemotherapeutic agents. The AuNPs as delivery systems can also promote tumor accumulation and interactions with cellular components through passive targeting mediated by the EPR effect. The preference for AuNPs over organic NPs (liposomes) is motivated by their ability to escape systemic clearance by RES, while their counterparts can be excreted and biodegraded before they reach their target sites [74,166]. As such, aptamer-targeted AuNPs-based systems could be explored to confer specificity and enhance the activity of chemotherapeutics through synergistic effects. The synergistic effects could be due to the combined activities of the drug and AuNPs core as well as aptamers. 

**Figure 3:**
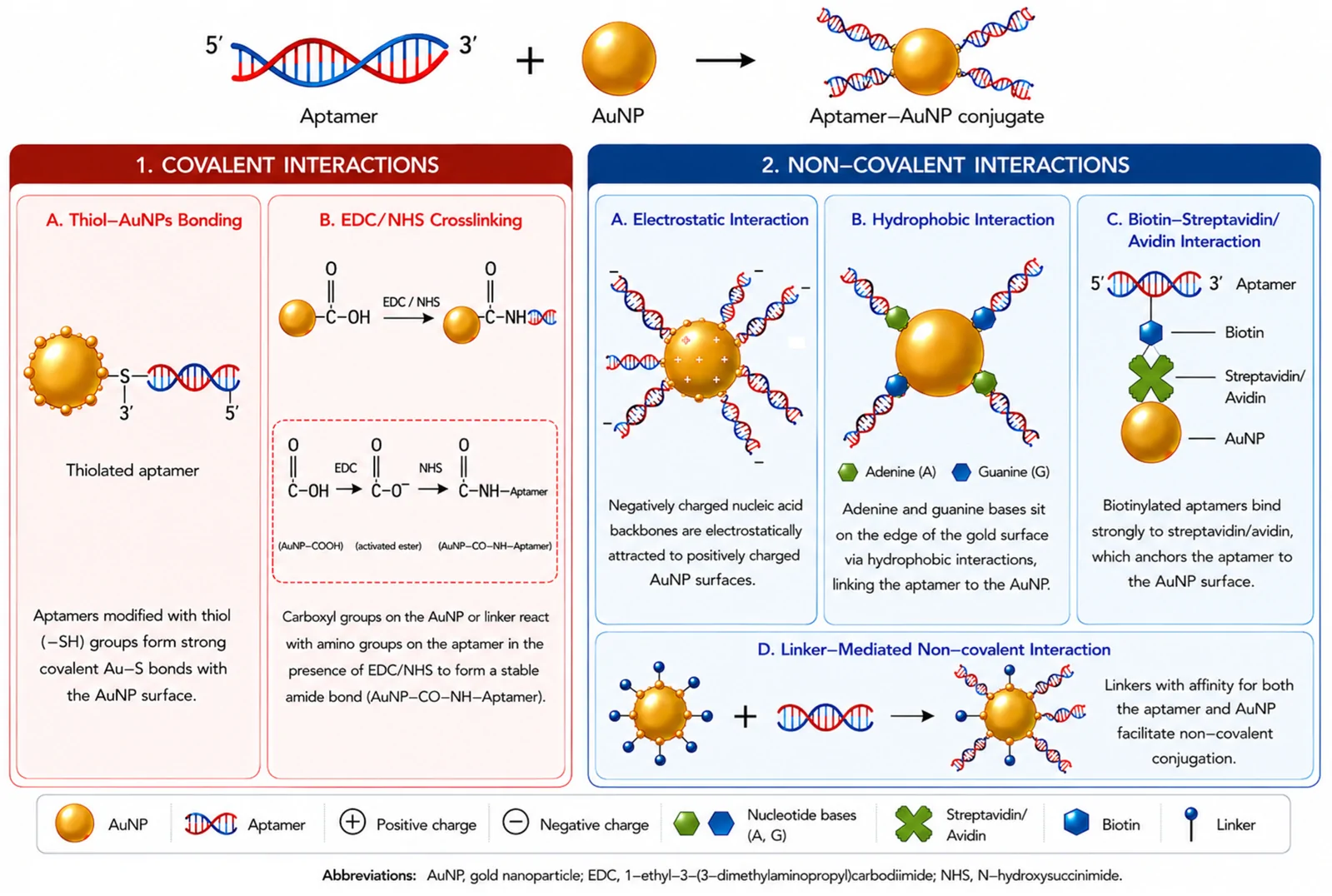
Overview of conjugation strategies used to couple CRC aptamers to AuNPs. Covalent and non-covalent chemistry can successfully be used to functionalize AuNPs with aptamers. The image was created using ChatGPTPlus (GPT-5.6 Sol; OpenAI, San Francisco, CA, USA).

The conjugation of aptamers to AuNPs relies on covalent and non-covalent interactions using the chemistries highlighted in Figure 3. Covalent conjugation is achieved via thiol-gold bonding or through crosslinking using 1-ethyl-3-(3-dimethylaminopropyl)carbodiimide/Nhydroxysuccini-mide (EDC/NHS). Thiol-gold bonding entails the attachment of thiolated molecules to AuNPs. Hence, aptamers that are modified with thiol-containing molecules could form strong covalent bonds with the AuNP surface [167]. The EDC/NHS linkage is created by reacting AuNPs with a carboxylic acid surface with aptamers containing an amino group end, thereby resulting in a stable amide bond [168].

The non-covalent conjugation of aptamers to AuNPs can occur through electrostatic and hydrophobic interactions primarily via the spontaneous adsorption of aptamers onto the AuNP surface. Electrostatic conjugation is driven by interactions between the negatively charged nucleic acid backbone and positively charged AuNP surfaces. On the other hand, the hydrophobic conjugation of aptamer to AuNPs utilizes non-covalent physical adsorption driven by attractive forces between hydrophobic nucleotide bases and the AuNP surface. Unmodified single-stranded DNA or RNA aptamers interact with the AuNPs surface via nitrogenous bases (especially adenine and guanine rings) through hydrophobic interaction. Non-covalent bonds can also be formed through streptavidin–biotin interactions or the use of linkers that facilitate interaction between the aptamer and AuNPs. However, the most common conjugation strategy of aptamers to AuNPs is direct covalent bonding, especially through thiol–AuNPs interaction [95].

In CRC therapy, biomarkers serve as useful targets for binding with aptamers, where upon binding, the aptamers then direct the AuNPs to the tumor site. The feasibility of this strategy has been demonstrated in several studies (Table 5). In a study by Go et al., a cellular prion protein (PrPC) aptamer conjugated to AuNPs and dox (PrPC-Apt-DOA) greatly increased dox uptake into SNU-C5 CRC cells, reduced cell proliferation and significantly increased cell death through apoptosis in comparison with free dox treatment, as shown in PrPC-Apt-DOA [169].

As photothermal agents, hollow AuNPs functionalized with AS1411 aptamers and C26 colon cancer cell membrane have been used for the targeted delivery of methotrexate to C26 colon cancer cells. The AuNPs-AS1411 aptamers were injected into the mice, which was followed by near-infrared irradiation after 24 h. The AuNPs were further tested for cytotoxic and photothermal activity in C26 cells compared to CHO cells, which do not express the target protein. The AuNPs-AS1411 aptamers caused a significant decrease in tumor volume, less toxicity to CHO cells, and reduced damage to the kidneys, liver and spleen in tumor-bearing mice [170]. MUC1 aptamers were used to deliver SN38-conjugated hyaluronic acid-loaded AuNPs targeted against MUC1-positive cells (metastatic colon cancer SW480 cells, HT29 cells) and MUC1-negative (CHO) cells. After laser irradiation, the treatment showed higher cytotoxicity and suppressed the migratory potential in MUC1-positive cells compared to CHO cells, highlighting the potential of aptamer-AuNPs in the photothermal therapy of CRC [77]. The anticancer properties demonstrated by these studies indicate the practicality of aptamer-targeted AuNPs-based systems for CRC treatment. Therefore, we propose that aptamer-targeted AuNPs-based systems could confer specificity, reduce off-target toxicity, as well as enhance activity through synergistic effects when used in the treatment of CRC.

**Table 5:** Aptamers-AuNPs strategies used in CRC treatment.

Aptamer	Target Biomarker	AuNPs Size and Shape	Functionalization Approach	Treatment Outcomes	References
MUC-1 aptamer	MUC-1	24 nm AuNPs	Conjugation to AuNPs through thiol-modified aptamer	Laser irradiation caused higher cytotoxicity in HT-29 cells compared to the control	[167]
PrPC aptamer	Cellular prion protein	20 nm AuNPs, spherical	Conjugation of aptamer through thiol binding	Caused selective uptake of dox and decreased cell proliferation in SNU-C5 CRC cells through apoptosis	[169]
AS1411	Nucleolin	50 nm AuNPs, Hollow	Covalent bonding via EDC/NHS amide linkage	Efficiently delivered methotrexate to C26 CRC cells while sparing nucleolin-negative CHO cells, laser-irradiated AuNPs caused higher cytotoxicity and reduced tumor volume in comparison to control	[170]
EpCAM aptamer	EpCAM	40 nm AuNPs	Covalent attachment of aptamer to carboxylic group of PEG through EDC/NHS activating agent	Elicited selective delivery of 5-FU to HT-29 cells, increased apoptosis and inhibited tumor growth in C57BL/6 mice bearing HT-29 tumors	[171]
MUC-1 aptamer	MUC-1	75 nm AuNPs, Spherical	Covalent attachment of aptamer through EDC/NHS linkage	Increased internalization of SN-38 into HT29 and SW480 CRC cells, laser-irradiated AuNPs increased cytotoxicity in target cells but not CHO control cells	[77]

## 6. Safety of Aptamer-Targeted AuNPs-Based Systems

**Figure 4:**
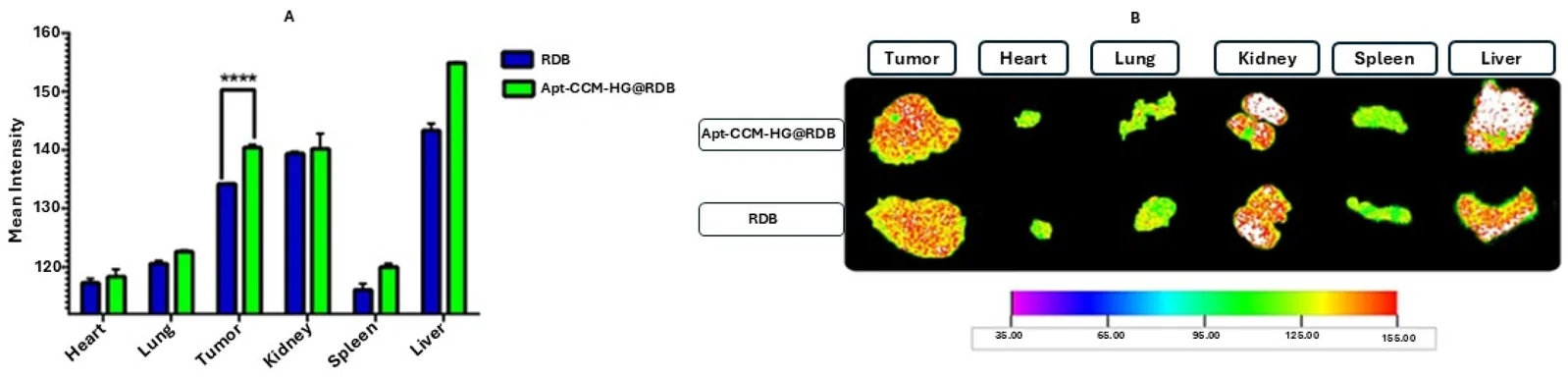
(**A**) Biodistribution of free RDB or Apt-CCM-HG@RDB in major organs of mice after 24 h post injection via tail vein. (**B**) Fluorescence analysis was used to quantify accumulation of the treatments in various organs. RDB represents rhodamine blue and Apt-CCM-HG represents apt + cancer cell membrane + hollow AuNPs. Reprinted with permission from Elsevier [170].

**Figure 5:**
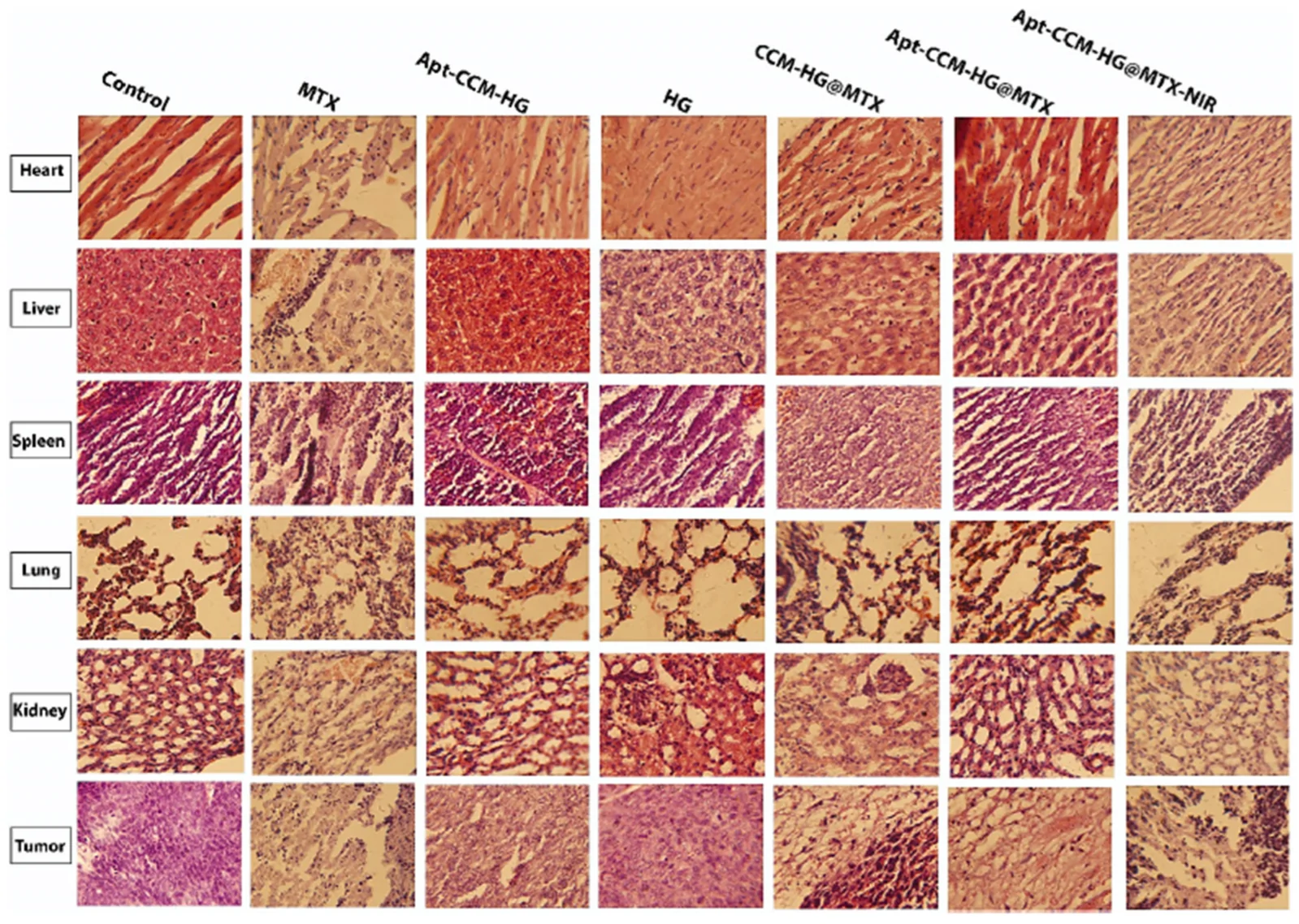
Effect of treatments on various tissues harvested 21 days post-administration via tail vein injection. The tissues and tumor harvested from C26 BALB/c mice 21 days post-administration were stained with hematoxylin and eosin and analyzed for any pathological features. MTX—methotrexate, CCM—cancer cell membrane, HG—hollow gold nanoparticles, Apt—aptamer. Reprinted with permission from Elsevier [170].

Current research about aptamer-targeted AuNPs continues to show promise in the management of CRC. Preclinical studies by Hassibian et al. (2024) examined the biodistribution of aptamer-targeted AuNPs in the liver, heart, kidney and spleen of female BALB/c mice after intravenous administration of a single dose [170]. The fluorescence imaging (Figure 4) demonstrated a higher accumulation of aptamer-targeted AuNPs in tumor cells compared to the control. Pathological examination after 21 days of treatment revealed notable liver toxicity between animals administered free methotrexate and aptamer-methotrexate-loaded AuNPs (Figure 5) [170]. Hence, this study suggested that aptamer-targeted AuNPs could be safe and serve as prospective tools for the clinical therapy of CRC. To our knowledge, no clinical trial of aptamer-based AuNPs in CRC has been conducted. Hence, extensive studies are warranted to evaluate the safety and efficacy of this platform in both animals and humans.

## 7. Challenges and Future Perspectives

Aptamers have shown promise in the targeted treatment of CRC due to their effectiveness and selectivity. Nevertheless, their possible translation to clinical use faces significant challenges and limitations. These include inadequate experimental methods, limited long-term toxicity information and insufficient clinical data. Addressing these issues will be helpful in harnessing the full potential of aptamer-targeted AuNPs in CRC therapy.

Aptamers are susceptible to rapid renal filtration and nuclease-mediated cleavage, and this could impede their clinical translation. Aptamer qualities can be improved through chemical modification or using carriers [172]. The susceptible moieties of the oligonucleotide are the sugar ring, phosphodiester backbone and the terminal ends. Hence, the sugar backbone could be substituted at 2′-O-methyl by 2′-fluoro group or modified by phosphorothioate. Capping the 3′ end with inverted thymidine has also been utilized in pegaptanib derivatives resulting in ARC1779 and ARC1905, which prevented nuclease degradation [125,173]. Likewise, spiegelmers, which are mirror images of aptamers, conferred an evasion of recognition by nucleases, and this approach has been successful in NOX-A12 RNA aptamer [174]. However, chemical modification increases the synthesis cost and could cause undesirable immunological responses. Additionally, carriers or delivery systems have been used to protect aptamers from degradation and enhance their bioactivity. The use of bulky moieties such as cholesterol, serum albumin, polyethylene glycol, and other nanocarriers has been reported to improve aptamer biodistribution and prolong their circulation time. However, biocompatibility and toxicity issues limit their application [174]. MNPs, particularly AuNPs, have increased aptamer resistance to renal filtration, promoting stimuli-responsive release and controlled activity [125,172].

In addition, most studies on aptamer-targeted AuNPs rely on in vitro assays performed on CRC cell lines and a few animal studies with inadequate sample size. Moreover, many in vitro studies do not consider the tumor microenvironment. To gain insight into the pharmacokinetic and pharmacodynamic parameters of aptamer-targeted AuNPs, extensive studies into dose–response assessments, safety, genotoxicity, adverse events, and the long-term toxicity of aptamer-targeted AuNPs needs to be intensified. This will ensure that the required regulatory requirements are met for translation into approved therapeutics for clinical management of CRC.

Aptamers targeted at different stages (I–IV) can be explored to treat CRC. Various biomarkers are involved in CRC progression. For example, β-catenin is responsible for tumor initiation and recurrence, Kirsten rat sarcoma virus (KRAS) promotes tumor growth in early stage cancers, programmed cell death 4 is implicated in stage II of CRC, KRAS and serine–threonine protein kinase implicated in tumor progression are found in stage III, while phosphatase and tensin homolog (PTEN), which promotes drug resistance and tumor metastasis, is implicated in CRC stage IV [72]. Therefore, an exploration of aptamers with strong affinity for these stage-specific markers could improve the therapeutic outcomes and overall survival of CRC patients. Additionally, current immunotherapies could be improved by replacing antibodies with aptamers, where their success could yield cheaper generics that will be cost-effective for the public.

## 8. Concluding Remarks

CRC remains a public health challenge and is a leading cause of cancer-associated morbidity and mortality. The disease has poor prognosis due to the lack of effective treatment options and a low response rate to current chemotherapeutic drugs. Chemotherapy as a treatment modality lacks specificity, leading to toxic effects that reduce therapeutic efficacy and survival rate. Therefore, it is imperative to find more effective treatment strategies that can improve the prognosis of CRC. AuNPs have been used in disease therapy owing to distinctive characteristics such as biocompatibility and multiple bioactivities. Through passive targeting, they localize efficiently into tumor cells but also internalize into off-target organs, leading to adverse effects. Hence, active targeting using aptamers can be exploited for CRC therapy. An aptamer-AuNPs-based strategy could facilitate an efficient delivery of drugs to CRC cells, which could reduce unwanted systemic effects, decrease the development of multidrug resistance to chemotherapeutics and enhance treatment outcomes of CRC.

Aptamers have shown potential for the development of innovative anticancer strategies owing to distinctive features such as their small size, rapid tissue penetration, low immunogenicity and high target specificity. A strategy that combines aptamers and AuNPs will confer aptamer stability as well as ensure drug specificity and efficacy through synergistic effects. Although aptamer-targeted research is ongoing, there is still a paucity of research regarding the exploration of aptamer-targeted AuNPs-based systems for chemotherapeutic and photothermal applications. Hence, research should be intensified to encourage the clinical translation of this strategy from bench to bed. Furthermore, extensive animal and clinical trials should be conducted to ascertain the safety, efficacy, and pharmacokinetics of these conjugates in order to understand the absorption, distribution, metabolism and excretion of aptamer-targeted AuNPs in humans.

## Figures and Tables

**Table 1:** Nanomedicines in clinical trials for cancer therapy.

Name	Composition	Indication	Clinical Trial Phase	Clinical Trial Identifier
THE 001	Thermosensitive liposome loaded with dox	Treatment of metastatic soft tissue sarcoma	Phase 1	NCT05858710
CPX-1	Liposomal NPs combining irinotecan and floxuridine	Treatment of advanced CRC	Phase 2	NCT00361842
Aurimmune (CYT-6091)	PEGylated colloidal AuNPs targeted at TNF-α	Treatment of advanced solid tumors	Phase 1	NCT00356980
V940	Lipid NPs loaded with mRNA	Cancer vaccine against bladder cancer, melanoma, and renal cancer	Phase 1	NCT03313778
BIND-014	PSMA-targeted polymeric NPs loaded with docetaxel	Treatment of advanced cancers including CRC, prostate, non-small lung cancer, head and neck cancer	Phase 1	NCT01300533
CALAA-01	Polymeric NPs targeted against transferrin receptors	Treatment of CRC and other solid tumors	Phase 1	NCT00689065

**Table 2:** AuNPs-based therapies and their outcomes in CRC cell lines.

AuNPs-Based Therapy	Cancer Model	Outcome	Reference
Spherical AuNPs–cetuximab conjugate	Colon cancer (HT-29) cells	Increased cytotoxicity and apoptosis in cells compared to free drug and AuNPs alone	[21]
Spherical AuNPs loaded with 5-FU	Colorectal cancer cells	Induced cell death and stopped cell cycle progression in cells compared to free drug	[92]
Spherical AuNPs loaded with dox	Colon cancer (SW620) tumor-laden female BALB/c nude mice	Decreased cell viability and reduced tumor volume compared to control	[93]
Spherical AuNPs functionalized with quercetin	Colon cancer (SW620) tumor-bearing female BALB/c nude mice	Enhanced cytotoxicity of SW620 cells and significantly reduced tumor volume in tumor model in mice	[90]
Spherical AuNPs synthesized with *Enterococcus* sp.	Colon cancer (HT-29) cells	Induced cytotoxicity compared to untreated control	[96]
Spherical AuNPs conjugated with regorafenib	Colon cancer (HCT-116) cells	Increased cytotoxicity and enhanced apoptosis in CRC cells compared to free regorafenib	[97]
Spherical AuNPs loaded with curcumin	Colon cancer (HT-29) cells	Induced cytotoxicity compared to untreated control	[98]
Anti-EGF receptor (EGFR) gold nanorods loaded with 5-fluorouracil	Human CRC (HCT 116 and HT-29) cells	Induced apoptosis and necrosis	[94]

## Data Availability

The data presented in this paper are extracted from articles already published in public domains and cited throughout the manuscript.
